# Invited commentary on Australian fetal alcohol spectrum disorder diagnostic guidelines

**DOI:** 10.1186/1471-2431-14-85

**Published:** 2014-04-01

**Authors:** Susan J Astley

**Affiliations:** 1 FAS Diagnostic & Prevention Network, Center on Human Development and Disability, University of Washington, Box 357920, Seattle, WA 98195-7920, USA

**Keywords:** Fetal alcohol spectrum disorders, FASD 4-digit diagnostic code

## Abstract

The publication of Australian fetal alcohol spectrum disorder (FASD) diagnostic guidelines marks an important step forward in Australia’s efforts to prevent FASD. But do we need yet another set of FASD guidelines? At the 5th International FASD Conference, the ever growing number of FASD diagnostic guidelines was identified as a core area of concern by leaders in FASD worldwide. All agreed we need to strive to adopt a single set of guidelines. It is essential that FASD diagnosis advance to incorporate new knowledge and technology. But to date, the field of FASD has seen multiple sets of guidelines published that do not address the important question-*How is the performance of these new guidelines superior to the performance of existing guidelines to warrant/justify their introduction into the medical literature*?

The Australian guidelines include FAS, PFAS and Neurodevelopmental Disorder-Alcohol Exposed (ND-AE). This latter group includes individuals with severe CNS abnormalities without the physical features of FAS. This is the group the 4-Digit-Code calls Static-Encephalopathy-Alcohol-Exposed (SE-AE). The criteria for FAS, PFAS, and ND-AE (or what the 4-Digit-Code calls SE-AE) are identical between the Australian and 4-Digit-Code guidelines with the exception of one very small, but very consequential difference in facial criteria for PFAS. The 4-Digit-Code requires a Rank 3 FAS facial phenotype for PFAS (*J Popul Ther Clin Pharmacol***20**(3)**:**e416–e467, 2013); the Australian guidelines relax the criteria to include the Rank 2 FAS facial phenotype. This relaxation of the criteria renders the facial phenotype NOT specific to prenatal alcohol exposure as confirmed in published empirical studies. If the facial phenotype is not specific to (caused only by) prenatal alcohol exposure one can no longer validly call the outcome PFAS. When one makes a diagnosis of FAS (full or partial), one is stating explicitly that the individual has a syndrome caused by prenatal alcohol exposure. One is also stating explicitly that the biological mother drank alcohol during pregnancy and, as a result, harmed her child. These are bold conclusions to draw and are not without medical, ethical, and even legal consequences. So the question remains-Why go against the published empirical evidence and relax the PFAS facial criteria into the normal range?

## 

The publication of Australian fetal alcohol spectrum disorder (FASD) diagnostic guidelines [1] marks an important step forward in Australia’s efforts to prevent FASD. Accurate identification of the full spectrum of outcome caused by prenatal alcohol exposure is central to FASD screening, diagnosis, intervention, surveillance, and ultimately prevention [2,3]. But do we need yet another set of FASD guidelines?

FASD diagnostic guidelines have evolved over time since the term FAS was first coined in 1973 [4]. Early guidelines were gestalt (purposely broad and conceptual) in nature and administered primarily by geneticists/dysmorphologists. The 1996 Institute of Medicine FASD guidelines [5] would be the last in this line of gestalt approaches to diagnosis. In 1997, the FASD 4-Digit-Diagnostic-Code was introduced to overcome the limitations (inaccuracies) of the gestalt method of diagnosis [6-8]. The 4-Digit-Code introduced an interdisciplinary approach guided by rigorously and empirically case-defined criteria. When the 4-Digit-Code was introduced into the medical literature, it was presented in the form of an empirical study demonstrating its superior performance to the gestalt approach it proposed to replace [7]. Over the next 17 years, it performance would continue to be extensively assessed (validated [2,9]) through MRI studies [10], population-based screening/surveillance studies [11-14], and patient follow-up surveys [15]. Between 2004 and 2013, five additional FASD diagnostic guidelines will be introduced into the literature [16-19]. All proposed an interdisciplinary approach using defined criteria. Most were established through a consensus process, but none had performance assessed (validated) prior to or even after publication. Most present with criteria that are marginally different from the 4-Digit-Code, but none provide empirical evidence demonstrating their superior performance relative to existing guidelines. It is essential that FASD diagnosis advance to incorporate new knowledge and technology. But to date, the field of FASD has seen multiple sets of guidelines published that do not address the important question-*How is the performance of these new guidelines superior to the performance of existing guidelines to warrant/justify their introduction into the medical literature*[2]? At the 5th International FASD Conference in 2013, the ever growing number of FASD diagnostic guidelines was identified as a core area of concern by leaders in FASD worldwide. All agreed we need to strive to adopt a single set of guidelines.

The most recent guidelines introduced into the literature are the Australian guidelines [1]. More accurately, they are consensus recommendations providing a foundation for development of Australian FASD diagnostic guidelines. The Australian guidelines adapted elements of the 4-Digit-Code and Canadian Guidelines. Let’s take a closer look.

The Australian guidelines include FAS, PFAS and Neurodevelopmental Disorder-Alcohol Exposed (ND-AE). This latter group includes individuals with severe CNS abnormalities without the physical features of FAS. This is the group the 4-Digit-Code calls Static-Encephalopathy-Alcohol-Exposed (SE-AE). The Australian guidelines chose not to use the term Alcohol-Related-Neurodevelopmental-Disorder (following the 4-Digit Code and DSM-5 [20] conventions) due to the implication of causality between exposure and outcome that cannot be confirmed. The criteria for FAS, PFAS, and ND-AE (or what the 4-Digit-Code calls SE-AE) are identical between the Australian and 4-Digit-Code guidelines with the exception of one very small, but very consequential difference in facial criteria for PFAS. The 4-Digit-Code requires a Rank 3 FAS facial phenotype (“2.5” of the 3 facial features must be present) for PFAS; the Australian guidelines relax the criteria to include the Rank 2 FAS facial phenotype (2 of the 3 facial features). This relaxation of the criteria renders the facial phenotype NOT specific to prenatal alcohol exposure as confirmed in published empirical studies [2,3,21,22]. In one of those studies 25% of a group of high-functioning (mean IQ 120) children with confirmed absence of prenatal alcohol exposure presented with 2 of the 3 features. This study clearly demonstrated that the PFAS facial phenotype proposed in the Australian guidelines is not observed exclusively among children damaged by prenatal alcohol exposure. If the facial phenotype is not specific to (caused only by) prenatal alcohol exposure (and we already know the growth and CNS abnormalities are not caused only by prenatal alcohol exposure) one can no longer validly call the outcome PFAS. This problem is not resolved by requiring a confirmed prenatal alcohol exposure. The problem lies in the name (PFAS) given to the condition. When one makes a diagnosis of FAS (full or partial), one is stating explicitly that the individual has a syndrome caused by prenatal alcohol exposure. One is also stating explicitly that the biological mother drank alcohol during pregnancy and, as a result, harmed her child. These are bold conclusions to draw and are not without medical, ethical, and even legal consequences. If the growth, face and CNS criteria for PFAS are not specific to prenatal alcohol exposure, a clinical team is in no position to claim the child has a condition caused by their mother‶s alcohol use. A second problem arises with the relaxation of the facial features for PFAS into the normal range (requiring only 2 of the 3 features). If the facial criteria are relaxed, the Australian PFAS group is no longer clinically distinct from the Australian ND-AE group [2,3]. Note the only feature that distinguishes the Australian PFAS group from their ND-AE group is the facial criteria. But if the facial criteria for PFAS are relaxed into the normal range, the facial criteria are no longer clinically distinct from the facial criteria for ND-AE. If there is no clinically meaningful distinction between PFAS and ND-AE, what is the justification for creating two separate diagnostic subgroups? So the question remains-Why go against the published empirical evidence and relax the PFAS facial criteria into the normal range?

The Australian guidelines report the UW 4-Digit-Code could also be derived if desired“. Unfortunately, the only issue preventing clinicians from deriving a 4-Digit-Code that would be in complete compliance with the Australian guidelines is the relaxation of the PFAS facial criteria from a Rank 3 to a Rank 2. If the Australian guidelines required a Rank 3 face for PFAS, it would not only gain the specificity required to validate the use of the term PFAS, but the 4-Digit-Codes would derive 4-Digit clinical categories (FAS, PFAS, SE-AE) that match the Australian clinical categories (FAS, PFAS, ND-AE). If the 4-Digit-Code said it was FAS, so would the Australian guidelines. If the 4-Digit-Code said it was PFAS, so would the Australian guidelines. And if the 4-Digit-Code said it was SE-AE, so would the Australian guidelines (but the Australian guidelines would simply give it a different label (ND-AE)). Individuals who present with moderate CNS dysfunction (what the 4-Digit-Code calls Neurobehavioral-Disorder-Alcohol-Exposed) would receive 4-Digit-Codes documenting their outcomes, but in accordance with the Australian guidelines would not receive a label. This would seem a reasonable interim solution as Australians further assess how to handle this moderate end of the FASD spectrum. The authors report “*Although there is an extensive evidence base confirming prenatal alcohol exposure causes the full range of outcomes from moderate to severe CNS dysfunction and a growing evidence base documenting significant CNS structural abnormalities among alcohol-exposed individuals with moderate dysfunction; panel members identified the need for additional evidence to more fully evaluate the validity of diagnosis based on moderate CNS dysfunction*” Rendering a 4-Digit-Code would allow one to quickly apply a label retroactively in the event Australia elects to recognize (e.g., label) this moderate end of the FASD spectrum in the future.

## Response

Rochelle E Watkins, Elizabeth J Elliott, Janet M Payne, Colleen M O’Leary, Jane Halliday, Jane Latimer, Amanda Wilkins, Raewyn C Mutch, James P Fitzpatrick, Heather M Jones, Lorian Hayes, Heather D’Antoine, Sue Miers, Elizabeth Russell, Lucinda Burns, Anne McKenzie, Maureen Carter, Carol Bower.

We have recently published recommendations for the diagnosis of fetal alcohol spectrum disorders (FASD) in Australia. These recommendations seek to address the known issue of underdiagnosis through supporting improved awareness of FASD and increased national capacity to respond to, and ultimately prevent, these disorders. We used an adaption approach to guideline development in recognition of existing diagnostic guidelines. Recommended national standard diagnostic criteria for Australia are based on the University of Washington (UW) 4-Digit Diagnostic Code and Canadian guidelines for FASD diagnosis. Our conclusions emphasise the importance of evaluation to improve the evidence-base for policy and practice.

In her commentary on our recommendations, Astley has raised important questions about the publication of this work, including whether we need ‘yet another guideline’, and expressed concern about the recommended criteria for the diagnosis of partial fetal alcohol syndrome (PFAS). Guideline development is a recognised and widely used mechanism to influence clinical practice. The lack of guidelines for diagnosis in Australia has been identified as one of the factors contributing to the underdiagnosis of FASD. We sought to establish the basis for a standard national approach to diagnosis in Australia through the adoption or adaption of existing guidelines.

Existing diagnostic guidelines lack agreement on all aspects of diagnosis, and the recommended diagnostic criteria for PFAS remain subject to debate. Although our recommended diagnostic criteria for PFAS in Australia based on the presence of 2 characteristic facial features differ from the UW guidelines, they lie within the range of criteria recommended by other guidelines. We sought peer review and publication of our findings to provide transparency and promote awareness of our work nationally and internationally. We support international collaboration to develop a common approach to diagnosis, and believe that this process will strengthen the evidence base for action globally.

### Background

North America leads the world in the production of guidelines for the diagnosis of fetal alcohol spectrum disorder (FASD), and these provide a rich resource for those seeking to address and prevent the harm caused by prenatal exposure in other contexts. We recently published Australian recommendations for FASD screening and diagnosis [1] with the aim of supporting clinical decision-making to improve the identification, management and prevention of these disorders based on a standard national approach. In her commentary on our recommendations, Astley has raised some important questions about the need for these guidelines, the publication of this work and the recommended criteria for the diagnosis of partial fetal alcohol syndrome (PFAS).

#### National focus

The development of clinical practice guidelines has been identified as a critical activity at the national level to facilitate the delivery of effective health services [23]. The majority of published guidelines for the diagnosis of FASD were commissioned by government health agencies and motivated by concern about the impact of prenatal alcohol exposure at a national level. It is unlikely to be coincidental that North America has both published the greatest number of guidelines for diagnosis and gained recognition as leading the world in many aspects of FASD practice and research.

Guidelines are developed with the aim to influence practice. They need to be locally appropriate and integrated with strategies to facilitate their implementation. High quality guidelines developed at the international level can facilitate the development of locally appropriate guidelines for specific practice settings and, increasingly, local health providers are developing methods to integrate evidence in their own settings [24]. The need to improve service delivery and support health professionals’ capacity to diagnose FASD in Australia [25,26] underpinned the Australian Government’s call for development of a diagnostic instrument for Australia. The absence of guidelines for FASD diagnosis in Australia has been linked to underdiagnosis [27] and inaccurate FASD prevalence estimates [28]. The lack of a national standard approach undermines the effectiveness of interventions to educate health professionals, increase diagnostic and management capacity, and conduct surveillance and evaluation.

#### Adoption or adaption

Through the development of national recommendations for FASD screening and diagnosis we aimed to improve awareness of FASD and strengthen national capacity to identify, address and prevent the harms associated with alcohol consumption in pregnancy. Four key factors framed our approach: i) that there are no international consensus criteria for diagnosis; ii) that there is variation in diagnostic practices internationally [29]; iii) that the most recent guidelines were published in 2005, and none with a published formal review; and iv) that there was little evidence to support the natural emergence of any standard national approach to diagnosis in Australia.

A range of existing guidelines have been used for diagnosis in Australia. The Institute of Medicine criteria [5] were used in the first national surveillance study in 2001 [30], the Canadian guidelines [17] were used in the first national prevalence study in 2009 [31], and the University of Washington (UW) 4-Digit Diagnostic Code [8] has been proposed for national use by authors examining the issues of diagnostic capacity and surveillance [27,28].

We used an adaption approach to development [32] in recognition of existing guidelines for diagnosis, and our work followed the accepted process of identifying whether existing guidelines would be adopted or adapted for local use [33] within a recognised research framework [34,35]. We found no clear support for the adoption of any single existing guideline [1,26]. Our recommendations have been developed to provide the foundation for a coordinated national approach to service provision, and are based on the cornerstone contributions of existing diagnostic guidelines that have helped to advance practice globally.

#### Process and judgement

There are considerable challenges in producing evidence-based guidelines for FASD screening and diagnosis, and disagreement between guidelines can arise for various reasons, not all of which imply that different recommendations are invalid [36]. We developed recommendations for Australia based on the deliberations of a range of stakeholders within a consensus-based approach. As is often the case in guideline development, there was a recognised need to move beyond the limited research evidence [37], and rarely ‘an abundance of evidence available that leads directly to an indisputable recommendation’ [38] p. 35. Recommendation development is a largely qualitative process with the need to consider multiple factors and considerable potential to encounter differences in judgement for a number of reasons, including perceptions about relevance, feasibility, risks and benefits. Panel members noted the need for additional evidence to evaluate differences in recommendations between existing guidelines, as well as the recommendations developed for Australia [1].

In the specific case of differences between the UW and Australian recommendations with respect to the facial features required for a diagnosis of PFAS, the panel recognised that the criteria for the diagnosis of PFAS remain subject to debate [39,40]. Diagnosis of disorders within the FASD spectrum is consistently challenged by an insufficient understanding of factors which contribute to individual susceptibility and phenotypic variability, and research highlights both the clinical significance of facial abnormalities [41,42] and the potential for variation in facial dysmorphology [43,44]. We support the need for further research to examine the validity of recommended diagnostic criteria for PFAS and facilitate international consensus in this area, and believe it would be premature to conclude that the diagnostic category of PFAS based on the presence of 2 facial features is invalid at this time.

Recommended facial criteria for the diagnosis of PFAS in Australia are consistent with existing guidelines [17,18], and differences in categorisation between the UW and other guidelines do not result in substantial differences in outcomes such as a diagnosis on the spectrum where one would otherwise have not been made. With reference to the potential harm caused by a diagnosis of PFAS using the recommended Australian criteria, we do not claim causation in the case of PFAS when diagnosed based on the presence of 2 characteristic facial features. The use of a non-causal assumption could be considered more conservative than assuming causation where specificity is known to be less than 100%. Contrary to Astley’s comment, sufficient detail is recorded during the recommended assessment process to enable derivation of the 4-Digit Diagnostic Code despite differences between the diagnostic categories recommended in Australia and the UW guidelines. Further evaluation is critical to validating diagnostic criteria, and ensuring that the diagnostic categories used are based on meaningful distinctions across the spectrum.

We believe that our work provides a credible basis for advancing practice in Australia and, consistent with the purposes of the scientific literature and the need for transparency in recommendation development, we sought peer review and publication of a research paper which documents the methods used to develop these recommendations. If, through seeking peer review and publication to promote national and international awareness of our goals and progress, this process highlights broader issues that confront the development of FASD guidelines internationally, then we have achieved more than we had hoped.

## Competing interests

The author declares that she has no competing interests.

## Pre-publication history

The pre-publication history for this paper can be accessed here:

http://www.biomedcentral.com/1471-2431/14/85/prepub
